# Mesenchymal Stromal Cells, a New Player in Reducing Complications From Liver Transplantation?

**DOI:** 10.3389/fimmu.2020.01306

**Published:** 2020-06-19

**Authors:** Andrew Owen, Philip N. Newsome

**Affiliations:** ^1^National Institute for Health Research Birmingham, Biomedical Research Centre at University Hospitals Birmingham NHS Foundation Trust, University of Birmingham, Birmingham, United Kingdom; ^2^Department of Anesthesia and Critical Care, University Hospitals Birmingham NHS Foundation Trust, Birmingham, United Kingdom; ^3^Centre for Liver and Gastrointestinal Research, Institute of Immunology and Immunotherapy, University of Birmingham, Birmingham, United Kingdom; ^4^Liver Unit, University Hospitals Birmingham NHS Foundation Trust, Birmingham, United Kingdom

**Keywords:** mesenchymal, stem, stromal, cell therapy, transplantation, liver

## Abstract

In response to the global burden of liver disease there has been a commensurate increase in the demand for liver transplantation. However, due to a paucity of donor organs many centers have moved toward the routine use of marginal allografts, which can be associated with a greater risk of complications and poorer clinical outcomes. Mesenchymal stromal cells (MSC) are a multi-potent progenitor cell population that have been utilized to modulate aberrant immune responses in acute and chronic inflammatory conditions. MSC exert an immunomodulatory effect on innate and adaptive immune systems through the release of both paracrine soluble factors and extracellular vesicles. Through these routes MSC can switch the regulatory function of the immune system through effects on macrophages and T regulatory cells enabling a switch of phenotype from injury to restoration. A key benefit seems to be their ability to tailor their response to the inflammatory environment without compromising the host ability to fight infection. With over 200 clinical trials registered to examine MSC therapy in liver disease and an increasing number of trials of MSC therapy in solid organ transplant recipients, there is increasing consideration for their use in liver transplantation. In this review we critically appraise the potential role of MSC therapy in the context of liver transplantation, including their ability to modulate reperfusion injury, their role in the reduction of medium term complications in the biliary tree and their potential to enhance tolerance in transplanted organs.

## Introduction

Although the global burden of liver pathology is often underestimated due to limitations in mortality recording systems in many countries (1), it is still estimated that over 2 million liver-related deaths occur worldwide (2). Whilst there are many causes of liver disease, end stage liver disease represents a shared final pathway and once reached, the only curative treatment remains liver transplantation (3). With increasing numbers of patients on transplant waiting lists and fewer donor organs there has been a move toward the use of marginal donor organs so as to increase the pool of available organs for transplantation (4). This comes at a clinical cost though, despite improvements in patient selection. Specifically the prolonged warm ischemic time in a donation after cardiac death (DCD) liver transplant results in increased morbidity, mortality, critical care stay, and overall cost (5). The ability to increase the donor pool further by pushing the boundaries of ischemia, as well as reducing the need for toxic immunosuppression could lead to a reduction in complications and an increased number of organs available for transplantation. Mesenchymal stromal cells (MSC) may offer a novel cell therapeutic approach to impact on these negative squeal and potentially allow for expansion of the donor pool.

## Transplant Related Hepatic Injury

The process of liver transplantation includes a combination of both warm ischemia (organ at body temperature) and cold ischemia (organ perfused with cold preservative solution). The relative contribution of these processes during organ retrieval depends principally on the transport duration of the donor organ after cold preservation (6) and the donor liver source (7). Broadly speaking donor livers can come from one of three sources; a living donor, a brainstem dead/heart beating donor (donation after brainstem death, DBD) or a donor undergoing circulatory arrest/non-heart beating donor (donation after circulatory death, DCD). The consequences of the prolonged warm ischemia seen in DCD include greater generation of reactive oxygen species and a delayed adaptive immune response resulting in an injury pattern characterized by hepatocyte loss as opposed to the predominantly endothelial injury seen following DBD (8). Biliary complications are often seen following liver transplantation, however their incidence is different when comparing DCD and DBD with a greater incidence of ischemic cholangiopathy in DCD compared with more strictures and bile duct leaks seen in DBD (9). Liver ischemia and reperfusion injury represent a complex combination of pathologies with a variety of cell types involved and a number of pre-disposing factors related to both the transplant recipient and the donor organ. Ischemia and reperfusion injury are distinct but related pathologies often considered together as ischemia/reperfusion injury (10), with ischemic injury representing the primary damage to cells due to an interruption in organ perfusion and reperfusion injury representing the immunological response to the generation of reactive oxygen species and products of cell death following the re-establishment of organ perfusion.

**Figure 1:**
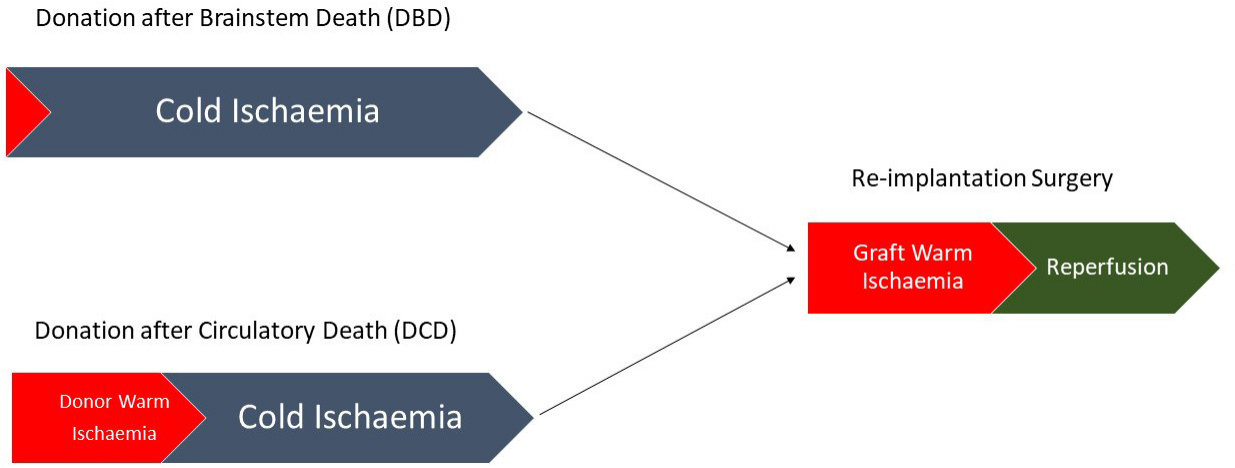
Pathway demonstrating the differences between DBD and DCD liver transplantation with no donor warm ischemic time seen in DBD compared with a variable amount of donor warm ischemia in DCD.

**Figure 2:**
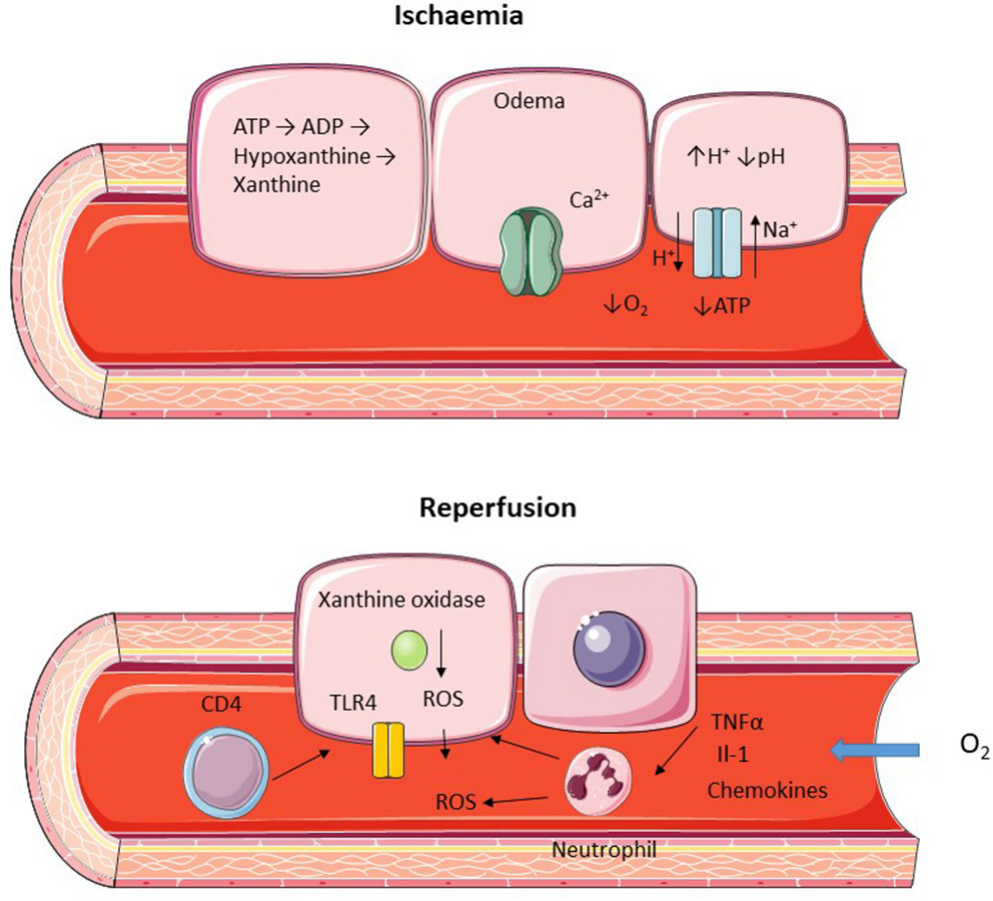
Diagram representing hepatic ischemic and reperfusion injury. Ischemia is characterized by a lack of oxygen supply leading to a depletion of ATP and cellular edema due to increased H^+^ leading to activation of the Na^+^/H^+^ transporter and inactivation of the ATP dependent Ca^2+^ pump. Reperfusion leads to restoration of O_2_ supply with ROS generation and recruitment of neutrophils and CD4^+^ lymphocytes.

Ischaemic injury occurs as a result of an inadequate oxygen supply to an organ and one of the first descriptions of liver ischemia was by Pringle in which his eponymous maneuver was shown to reduce bleeding by occluding liver vasculature (11). During liver transplantation warm ischemia can be further subdivided into donor warm ischemic time which is defined as the time from withdrawal of life support to the initiation of cold storage, and graft warm ischemic time which is defined as the time from removal from cold storage until reperfusion has occurred (12). There should be little donor warm ischemic time in living donors or DBD donors, whereas DCD donors will have a longer period of warm ischemia (Figure 1). The amount of cellular dysfunction seen following an ischemic insult is related to both the extent and duration of the period of ischemia (13). In humans oxygen is utilized in aerobic respiration in order to generate Adenosine Triphosphate (ATP), an energy source required for metabolic processes (14). Fermentation of glucose (often referred to in human physiology as anaerobic respiration) occurs when there is no oxygen present to act as the terminal electron acceptor leading to the generation of lactate, a less efficient process of energy production leading to the generation of H^+^ ions and the subsequent reduction in cellular pH. The subsequent H^+^ ion gradient leads to activation of Na^+^/H^+^ transporters in order to correct the intracellular pH generating an osmotic gradient and leading to cellular edema (15). Depletion of ATP also leads to inactivation of other ion pumps including the ATP dependent Ca^2+^ channels (Figure 2). There is also an increase in the breakdown products of ATP including xanthine and hypoxanthine (16). Hepatocytes are particularly vulnerable to warm ischemia (17) and whilst there is some debate over whether necrosis or apoptosis is the predominant factor in hepatocyte death, both modes of cell loss demonstrate mitochondrial dysfunction as a key problem (18–20). Cold ischemia, or preservation related injury, confers similar problems to that of warm ischemia, however there seems to be an increased effect on the sinusoidal endothelial cells whose death at reperfusion has been shown to be related to the duration of cold ischemia through platelet induced apoptosis (21).

Following restoration of blood and oxygen supply to the ischemic liver further damage occurs in the form of reperfusion injury. The generation of reactive oxygen species (ROS) is a key mechanism in this process, initially produced by intracellular xanthine oxidize in combination with resident specialized macrophages (Kupffer cells) and infiltrating polymorphonuclear cells (22). Activated Kupffer cells release pro-inflammatory cytokines which enhance neutrophil recruitment leading to further propagation of ROS release (23, 24). Following reperfusion sinusoidal endothelial cells express a number of adhesion molecules aiding the recruitment of infiltrating immune cells (25). T lymphocyte, in particular CD4^+^ cells, is a key mechanism of injury following reperfusion (Figure 2) and blocking CD4^+^ recruitment leads to a significant reduction injury (26). Activation of Toll Like Receptors (TLR) in particular TLR4 by damage associated molecular patterns (DAMP) has also been shown to be an important cause of reperfusion injury with reduced damage when this pathway is inhibited (27, 28).

## Mesenchymal Stromal Cells

Mesenchymal stromal cells (MSC) are a multi-potent progenitor cell type capable of tri-lineage differentiation and immunomodulation (29). The description of cells in the bone marrow able to perform a supportive role was postulated over a century ago, however more recent work has developed the understanding of the physiological role that MSC play in the bone marrow stem cell niche (30). MSC do not appear to be limited to the bone marrow however, with MSC populations described in umbilical cord (31), placenta (32), adipose tissue (33), dermal tissue (34), and dental pulp (35). Debate still remains in the literature as to what constitutes an MSC and whether they represent true stem cells (36, 37) with the early literature plagued by problems with heterogeneous cells types and isolation techniques. With the advent of cell sorting and the publication of minimal criteria in order to describe MSC some of the problems seen in the earlier literature have improved but comparison between MSC types can still be problematic (29, 38, 39). Whilst the functional role that MSC play is still not fully elicited their ability to modulate the immune system has been well described (40, 41). Variation in human MSC function has been variably ascribed to different batches, donor sources and donor age, however evidence is mixed and there is as yet no standardized source or donor demographic definition to inform cell therapy in clinical trials (42–47). It has also been demonstrated that adipose MSC derived from obese donors have reduced proliferative and differentiation ability (48). MSC tissue source may be important due to differing expression of tissue factor/CD142, as whilst MSC have low levels of MHC Class I and no MHC Class II expression (49), an innate immune response can be triggered following MSC infusion with source and passage being major determinants of tissue factor expression (50). This innate immune response can trigger thrombosis and lead to micro and microvascular complications. This is an important consideration when designing clinical trials as a cell therapy with low tissue factor expression may reduce the potential risks associated with MSC administration or enable steps to be made to mitigate these effects such as co-administration with heparin (51).

**Figure 3:**
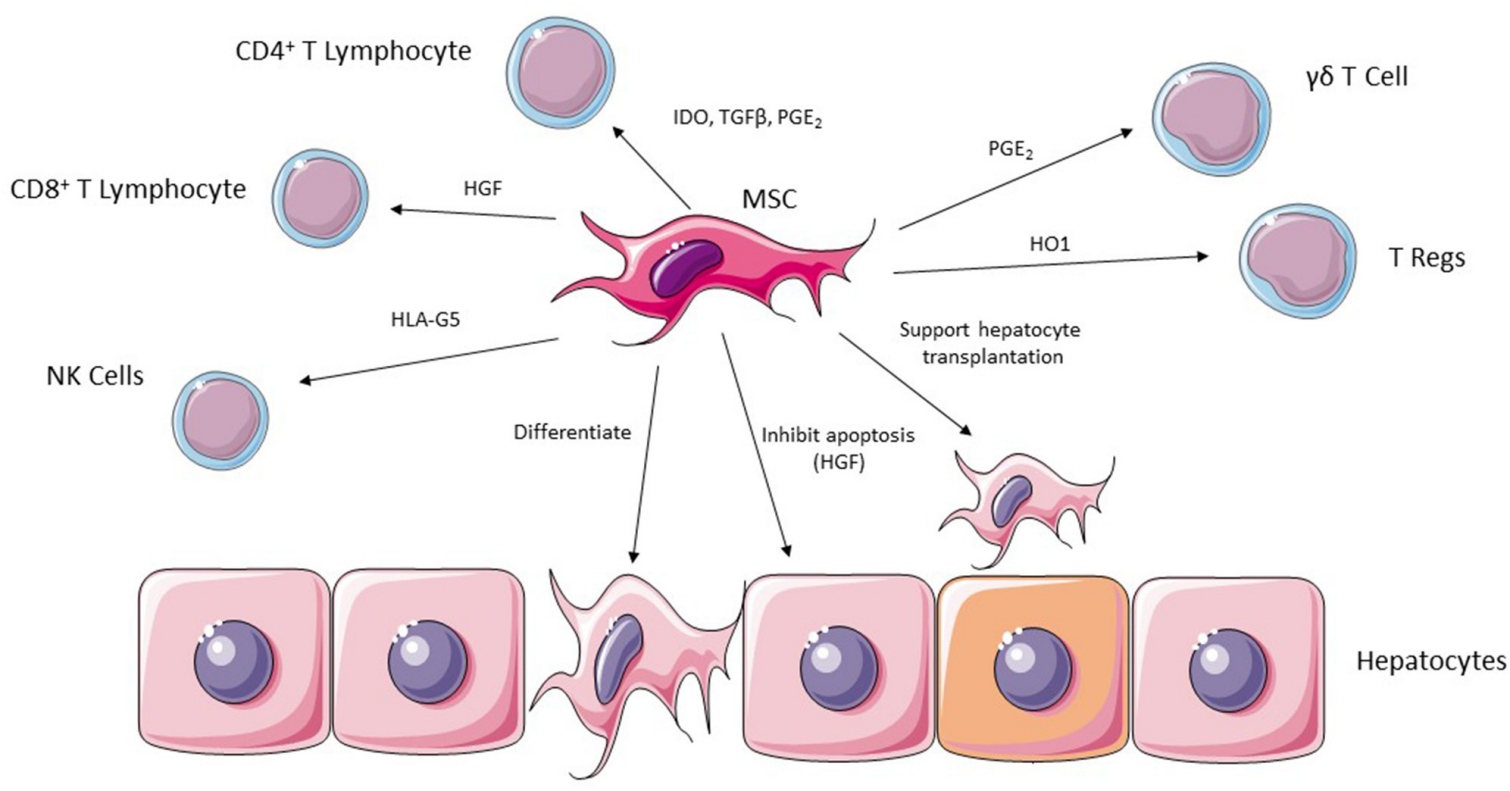
MSC are able to exert their effects in ischemic liver injury via a number of potential effector cells including CD4^+^ and CD8^+^ lymphocytes, NK cells, Regulatory T cells and γδT cells. They are also able to support hepatocyte transplantation into injured liver and can undergo differentiation into hepatocytes.

There have been a number of mechanisms described by which MSC are able to suppress the immune system with effects on both the innate and adaptive components. A key ability supporting the potential use of MSC in liver transplantation is their ability to suppress T cell activation and proliferation. Bone marrow derived MSC can upregulate the cyclin-dependent kinase inhibitor p27Kip1 and inhibit cyclin D2 leading to early cell cycle arrest in T lymphocytes (52). The effects on cyclin D2 are not limited to T cells however, with evidence that inhibition of B lymphocytes also occurs (53). MSC isolated from bone marrow can also reduce expression of MHC class I and II on dendritic cells, inhibiting their maturation and subsequent immune cell activation (54, 55). Another important ability of MSC is their effects on soluble factors, both secreting themselves and stimulating or inhibiting the secretion from other cells. Whilst many relevant mediators have been shown to be modulated by MSC therapy (Table 1; Figure 3), important factors include Il-10, TNFα, IDO, PGE_2_, and IFN-γ (56–62).

**Table 1:** Important cytokines involved in MSC immunosuppression.

**Cytokine**	**Effect**
C-C Motif Chemokine Ligand 2 (CCL2)	Suppress the activation and migration of Th17 cells
Haem Oxygenase 1 (HO1)	Suppresses T regulatory cell function
Hepatocyte Growth Factor (HGF)	Inhibits CD4^+^ and CD8^+^ T-lymphocyte function
Human Leucocyte Antigen G5 (HLA-G5)	Inhibits Natural Killer (NK) cells
Interleukin 6 (Il-6)	Inhibits neutrophil burst
Inducible Nitric Oxide Synthetase (iNOS)	Inhibits CD4^+^ T-lymphocyte function
Indolamine 2,3 dioxygenase (IDO)	Inhibits CD4^+^ and NK cell function
Nerve Growth Factor (NGF)	Binds to P75 on hepatic stellate cells and triggers apoptosis
Prostaglandin E_2_ (PGE_2_)	Inhibits CD4^+^ and NK cell function and inhibits differentiation of monocytes into myeloid cells and TNF production by dendritic cells
Transforming Growth Factor β (TGF-β)	Inhibits CD4^+^ T-lymphocyte function
TNF Stimulated Gene 6 protein (TSG6)	Switches macrophage phenotype to anti-inflammatory

Given that MSC appear to exert their effect by secreting soluble proteins and extra-cellular vesicles (MSC-EV) administration of the bone marrow MSC secretome may represent a potential therapeutic strategy avoiding some of the potential problems with the use of a cellular therapy (63). In particular, the ability of MSC-EV to transfer non-coding RNA may explain the prolonged effects seen after the rapid clearance of MSC from the systemic circulation (64).

There are a number of potential routes of administration for MSC and extensive research carried out in animal models has not revealed a consensus on the most efficient and effective route of delivery. MSC are relatively large cells compared with immune cells, and as such when administered systemically tend to be filtered by the lungs (65). Both intravenous and intra-arterial routes of administration have been described, however concerns have been raised regarding the potential risk of cerebral ischemia following intra-arterial administration of bone marrow MSC (66), although there are diseases where spread to cerebral tissue may be beneficial such as acute ischemic stroke (67). Subcutaneous administration may represent an alternative as there is evidence in graft versus host disease that bone marrow derived MSC can exert a remote effect when encapsulated (68), however these results have not been reproduced more widely. Intravenous administration is commonly used in clinical and pre-clinical investigation, however this can be problematic due to cells becoming trapped in the lung (69). Human bone marrow derived MSC have been demonstrated to express a number of adhesion molecules on their surface, and whilst many of these are lost in standard culture conditions, expression of CXCR4 is increased in hypoxic culture which may MSC homing to injured tissues (70, 71). Expression of CD44 has also been demonstrated to be involved in MSC engraftment to injured liver tissue *in* vitro suggesting direct administration or routes bypassing the lungs may lead to greater engraftment to the target organ (72). In large animal models of liver injury and liver fibrosis adipose tissue derived MSC have been injected via the intra-portal route directly into the liver vasculature and have demonstrated efficacy without an increase in intra-portal pressure, suggesting that this route is feasible and safe and would be directly accessible during a liver transplantation operation (73). Injection rate and needle gauge has also been shown to be an important consideration when administering MSC with optimum rates in a variety of cell types described *in vitro* (74).

MSC have been successfully used in a variety of different liver pathologies in both pre-clinical and clinical trials with no major safety concerns identified (75), rendering their potential use in liver transplantation a new and exciting prospect.

## MSC in Ischemia/Reperfusion Injury

Due to their ability to modulate both innate and adaptive immunity MSC represent an important potential therapy to ameliorate ischemia/reperfusion injury in liver surgery and transplantation. MSC have been shown to reduce ALT and IL-6 when administered systemically in models of hepatic ischemia reperfusion injury, but notably only if given before the ischemic insult (76). There also seems to be a reduction in TUNEL positive hepatocytes indicating less apoptosis in rat models of ischemia reperfusion injury following bone marrow derived MSC therapy (77). An important concern in the context of significant oxidative stress, as seen in both ischemia reperfusion injury and other forms of liver injury such as acute hepatic failure, is the ability of donor cells to survive in such a hostile environment. Bone marrow and adipose derived MSC have been shown to be resistant to oxidative stress and may themselves have antioxidant properties further suggesting a therapeutic role in these types of injury (78, 79).

Immune cell recruitment is a key feature in reperfusion injury with animal studies demonstrating that a reduction in injury can be achieved by blocking recruitment of both neutrophils and lymphocytes (26, 80). MSC derived from bone marrow can also reduce neutrophil recruitment and liver injury by enhancing the intracellular activation of p38 MPK phosphorylation leading to a decrease in the expression of CXCR2 on the surface of neutrophils as well as reducing the production of the neutrophil chemoattractant CXCL2 by inhibiting NK-κB p65 phosphorylation in macrophages (81). MSC-EV have also been shown to reduce liver injury if given systemically prior to an ischemic insult by reducing IL-6, IL10, TNFα, and IFN-γ levels as well as the number of caspase 3 positive apoptotic cells (82).

In larger animal models of ischemia reperfusion injury combined with partial hepatectomy, direct injection of MSC into liver parenchyma following surgery has been shown to reduce the number of apoptotic cells with a downregulation of Fas/Fas ligand and a reduction in enzyme activity of caspase 3, caspase 8 and caspase 9. There was also an increase in the apoptosis regulator protein Bcl2/Bax as well as a reduction in markers of autophagy such as Beclin1, ATG5, and ATG12 (83), suggesting an ability to regulate apoptosis in injured hepatocytes (84). Similar findings have been shown in small animal models of ischemia reperfusion injury (85). Topical application of adipose derived MSC can also augment liver regeneration following reperfusion injury with a reduction in necrotic areas and an increased number of regenerating cells (76) with activation of the notch2 pathway in MSCs (86).

## MSC Augment Hepatocyte Engraftment as an Alternative to Whole Organ Transplantation

Hepatic ischemia reperfusion injury can lead to significant hepatocyte loss with a subsequent loss of liver function. Over the last two decades transplantation of human hepatocytes as an alternative to whole organ transplantation has made significant progress with the development of protocols for isolation and storage of hepatocytes along with studies demonstrating early efficacy (87). A major draw-back of hepatocyte transplantation is the poor cell engraftment seen with only 0.1–0.3% hepatocytes remaining in the recipient liver (88). Co-transplantation of hepatocytes with fetal liver derived MSC has been shown to augment engraftment with a greater number of hepatocytes remaining in the recipient liver for a longer time along with improved liver function in animal models (89). This ability may as a result of MSC ability to provide tropic support to hepatocytes (90).

*In vitro* studies in human pancreatic tissue have shown that human bone marrow derived MSC can increase epithelial cell proliferation and neovascularisation (91) and more recent studies in human pancreatic islet cell transplantation have demonstrated that human bone marrow derived MSC are able to improve survival of transplanted islet cells in animal models (92). The ability of MSC to support transplanted pancreatic tissue via their effects on both vascularisation and endothelial support suggest an additional potential role in hepatocyte transplantation that warrants further study.

As an alternative to hepatocyte transplantation it has been argued that hepatocytes derived from MSC can replace injured cells and improve liver function. Certainly the engraftment and function of these cells appears to show benefit in animal models of bone marrow MSC (93–95), however there are conflicting results from pre-clinical studies rendering this a controversial area still requiring considerable attention before becoming a translatable therapy (96).

## Therapeutic Strategies Utilizing MSC in Liver Transplantation

Through their ability to reduce injury and cell death in models of ischemia and reperfusion injury it stands to reason that there is a role for MSC therapy in orthotopic liver transplantation (OLT). Coupled with this the ability of MSC to promote liver regeneration and reduce injury could lead to a further benefit in the post-transplant recovery period (75).

Work in small animal models has been encouraging, where adipose tissue derived MSC have been shown to reduce liver injury and TUNEL positive hepatocytes in rat models of liver transplantation (97). MSC derived conditioned media has also shown a beneficial effect in pre-clinical models of liver transplantation with a reduction in injury and an improvement in liver function when 50% reduced size transplantation is undertaken in rats with an increase in VGEF and MMP9 expression (98).

Indeed, a number of early phase clinical trials have been undertaken in this patient cohort with some encouraging results (99). Phase II clinical trials in patients undergoing liver transplantation have shown safety, but were not powered to demonstrate efficacy in patients receiving unrelated bone marrow derived MSC in the first few days following transplantation (100). MSC have also been tested for safety in a pilot study of patients with acute allograft rejection following liver transplantation. In patients treated with MSC in combination with standard immunosuppression protocols for acute rejection there was a significant increase in PGE_2_ and TGF-β1, as well as an significant increase in FoxP3^+^ T regulatory cells isolated from peripheral blood (101). The MYSTEP1 trial currently nearing completion may provide safety information on the use of donor derived bone marrow MSC in pediatric living donor liver transplantation, paving the way for more extensive study in this cohort (102).

Normothermic machine perfusion is rapidly becoming a therapy of interest in the preservation and regeneration of donated livers (103, 104). In contrast to static cold storage, the current gold standard for liver transplantation, normothermic machine perfusion involves cannulation and perfusion of an explanted liver at near physiologic conditions. This technique has advantages over cold storage techniques which tend to be poorly suited to steatotic grafts. With an interest in the use of more marginal grafts to increase the potential pool of donor organs reconditioning and regeneration of donor organs is an area of increasing interest. MSC represent a potential therapy to enhance the regeneration of donor liver tissue. Proof of concept for delivery of MSC during machine perfusion has recently been published paving the way for further study (105). MSC appear to be retained in the perfused liver and are still able to exert paracrine effects on liver tissue, similar findings have been demonstrated in porcine renal perfusion systems (106, 107). In renal perfusion, the effects of perfusion on MSC has been studied in both human and porcine bone marrow cells demonstrating that perfusion amplifies the negative effects of cryopreservation on MSC with lower levels of adhesion and an increase in MSC reactive oxidative species (107). Currently normothermic machine perfusion represents an interesting avenue and in combination with cell therapy may represent a new future standard in liver transplantation.

Another option for transplantation is the use of split liver grafts (108, 109), an accepted technique for pediatric transplantation, although a more mixed experience when splitting a donor liver between two adult recipients. By reducing ischemia/reperfusion injury at the time of surgery and promoting post-operative liver regeneration an increased number of patients could receive a transplantation if grafts were routinely split between 2 recipients. MSC offer a potential therapy to improve the outcomes in this type of liver transplantation surgery with some encouraging animal studies demonstrating reduction in TUNEL positive cells in rat models of partial hepatectomy following MSC treatment and improved survival in rat transplant models (110). MSC are also able to promote regeneration in a 30% partial liver transplant model by increasing the activity of AP-1 and NF-κB as well as demonstrating increased expression of cyclin D1 and proliferating cell nuclear antigen (PCNA) (111). Similar principles could be applied to hepatic resection/partial hepatectomy with improved regeneration allowing for greater resection and therefore increased likelihood of adequate margins in cancer surgery, and faster recovery times. So far animal work has supported this with MSC therapy increasing both hepatocyte and sinusoidal endothelial proliferation and recovery after partial hepatectomy in mouse models (112). MSC conditioned media can also promote liver regeneration in partial hepatectomy models (113). MSC have also been suggested as a rescue therapy for acute liver failure following partial hepatectomy. In rat models, bone marrow MSC are able to improve glucose metabolism and survival following 90% hepatectomy, possibly through modulation of the AKT/GSK-3β/β-catenin pathway (114).

In large animal models of 70% partial hepatectomy, MSC therapy has been shown to improve both liver and renal function, suggesting an initial benefit beyond liver regeneration (115). This improvement was through correction of hemodynamic dysfunction by increasing levels of platelet derived growth factor (PDGF) along with promoting regeneration in the kidneys by increasing pro regenerative cytokines.

## MSC as a Therapy in Post-Transplant Care

Following orthotopic liver transplantation, as with other organ transplantation, patients are required to take immunosuppressive agents in order to prevent the host immune system rejecting the graft. In most cases this is lifelong therapy which conveys a number of risks including infection and renal failure. Most strategies employ the inhibition of T-cell proliferation. By reducing or even eliminating immunosuppressant agents, morbidity in patients who have received a donor organ would be considerably reduced. MSC may offer an opportunity in these patients due to their ability to suppress T cell proliferation and activation.

Current consensus guidelines recommend the use of calcineurin inhibitors for maintenance immunosuppression in patients who receive a liver transplantation, supplemented with anti-metabolites or m-TOR inhibitors (116). With this in mind it is important to consider the effects of immunosuppressive drugs on MSC as well as the potential additive effect of MSC along with standard immunosuppressive agents used in liver transplantation. In the short term (<7 days) exposure of MSC from a variety of tissues to the calcineurin inhibitor tacrolimus, the anti-metabolite mycophenolate or the m-TOR inhibitor rapamycin, does not appear to lead to any detrimental effects. Prolonged exposure however leads to MSC toxicity in the case of tacrolimus, and reduced MSC proliferation in the case of mycophenolate and rapamycin (117). The combination of drugs seems to be important however with the effects of tacrolimus on MSC being reversed by the combined use of oxytocin (118). Whilst it has been demonstrated that avoidance of corticosteroids in patients following a liver transplantation is likely to be safe and reduce associated complications (119), corticosteroids are still widely used in the setting of liver transplantation, both for induction of immunosuppression and treatment of rejection (120). When combined with dexamethasone in *in* vitro assays the ability of MSC to suppress T cell proliferations seems to be reversed (121), however the ability of MSC to suppress natural killer cell activation seems to be enhanced with dexamethasone augmenting the ability of MSC to IL-2 and IL-12 induced CD69 expression and reduce production of IFNγ (122). This effect appears to be through the blocking of STAT1 in MSC by dexamethasone. MSC ability to suppress mononuclear cells appears to be enhanced by both dexamethasone and budesonide through increased IDO activity (123). In *in vivo* mouse models MSC derived from induced pluripotent cells do not seem to be effected by concomitant administration of dexamethasone (124). It would be challenging to test every combination of drugs that a patients could be taking when MSC therapy is administered, however the potential for both synergy and antagonism of medications with MSC therapy needs careful consideration when designing clinical trials in liver transplantation.

In rat models of orthotopic liver transplantation MSC are able to inhibit acute rejection by increasing expression of FoxP3 T regulatory cells (125), as well as prolonging survival by regulating the expression of TGF-β1 (126). In early phase clinical trials MSC have been shown to be safe in patients undergoing acute rejection following transplantation, as well as demonstrating an ability to increase TGF-β1 and prostaglandin E2 and increase the percentage of T regulatory cells present (101). In small animal models of cardiac and renal transplant allogeneic bone marrow MSC are able to induce organ tolerance by down-regulating T lymphocyte responses through expression of indolamine 2,3-dioxygenase (127, 128). In rats, both recipient and donor derived bone marrow MSC prolonged survival of transplanted livers through induction of FoxP3 T regulatory cells (125). Induction of transplant tolerance is a promising area of research but as yet has not made the transition into clinical studies.

### Challenges With MSC Therapy

Whilst MSC appear to show a great deal of promise in liver transplantation there are some challenges that need to be overcome.

#### Heterogeneity

MSC heterogeneity has long been a problem due to an inability to define and isolate a pure population of cells. The International Society for Cell Therapy (ISCT) defined minimal criteria for describing MSC in an attempt to overcome this but even with these in place the cell populations used still represent a heterogeneous population (29, 129, 130).

#### Immunogenicity and Haemocompatibility

Although MSC expression of MHC class II low/absent (49), haemocompatibility has still been demonstrated to be an issue, as MSC can upregulate class I and II MHC in response to interferon gamma (131). In mouse models allogeneic bone marrow derived MSC are cleared by the host immune system when transplanted into MHC class I and II mismatched recipients (132), and in graft versus host disease bone marrow derived MSC seem to generate an antibody response in a subset of patients (133). Despite these findings, MSC still seem to illicit a much slower response from the recipient immune system, coining the term “immune evasive” (56). Meta-analysis seems to suggest that whilst bone marrow derived cells appear to be safe, adipose tissue derived and perinatal tissue MSC carry a risk of acute thrombosis due to activation of the innate immune system (134–137). Problems due to haemocompatibility can be mitigated by pre-screening in clinical trials and has been suggested as a pre-requisite to cell therapy (50).

#### Oncogenesis

MSC have long been studied in oncology where resident MSC have been shown to support the tumor microenvironment and are seen as a therapeutic target to reduce cancer growth (138). It has been suggested that due to their ability to support growth and activation that MSC therapy is at risk of supporting or inducing tumor formation (139). Long term *in vitro* studies of murine bone marrow MSC have demonstrated the potential for oncogenic transformation (140), although this has been questioned in more recent studies which suggest there could have been cell line contamination (141). Whilst some small studies have demonstrated this concern may be valid, larger reviews of clinical trials do not support this hypothesis, however vigilance is still required as with all clinical trials (134).

## Clinical Trials

There has been an explosion in the use of MSC in clinical trials since the first study in humans carried out in 1995 (142), with MSC being one of the most clinically studied cell therapies worldwide (39). Due to the types of ischemia and reperfusion injury described earlier MSC represent a promising therapy in many areas liver transplantation and post-transplant care. Despite this, clinical trials in liver transplantation are still early on in their development with very few published trials. There have been few completed trials in patients undergoing liver transplantation but some still ongoing (Table 2), all are either phase 1 or phase 2 (145). This first published trials have demonstrated safety of MSC infusion in patients undergoing liver transplantation, but have also failed to demonstrate a benefit (100, 143). The effect of corticosteroids on patient's peripheral MSC populations has also been studied in the context of liver transplantation with a reduction in circulating MSC seen in those receiving corticosteroids (144). The implication of this finding is difficult to put into context though as there are clear differences between native and exogenous transplanted MSC in both number and properties. Interestingly this may be further proof of the pre-clinical studies suggesting that the MSC/HSC niche is controlled in part by the sympathetic nervous system (30). The ongoing MYSTEP1 trial is not only testing safety and efficacy of donor derived bone marrow MSC, but also the intra-portal route of administration in pediatric liver transplantation (102). Due to the inherent complexity and heterogeneity of clinical conditions, coupled with the variation in cell therapeutic products due to differences in donor, batch and cell tissue origin, the conduct and interpretation of clinical trials of MSC is complex and challenging. Whilst trials of MSC therapy in liver transplantation are still in their infancy, given the favorable safety profile demonstrated so far there is more potential work to be done in order to explore the role of MSC therapy in these patients.

**Table 2:** Ongoing or recently completed clinical trials with MSC in liver transplantation.

**References**	**Study description**	**Clinical Trial Registration**	**Patients**	**Status**	**MSC Dose**	**Key Findings**
Detry et al. (100)	Phase 1 trial testing MSC therapy in liver transplantation for safety and ability to induce tolerance	NCT01429038	19	Published	1.5-3 × 10^6^/kg IV	No side effects seen but tolerance not induced
Remuzzi, G	Test MSC ability to induce tolerance following liver transplantation	NCT02260375	20	Recruiting	1-2 × 10^6^/kg IV	Not published
Soeder et al. (143)	First in man case study of MAPC in patients following liver transplant (MiSOT-1)	NCT01841632	1	Published	1.5 × 10^8^ MAPC intra-portal	No acute complications seen
Sturm, E	Phase 1 trial to determine the safety of MSC in pediatric liver transplantation (MYSTEP1)	NCT02957552	7	Recruiting	2 doses 1 × 10^6^/kg intra-portal then IV	Not published
Walker et al. (144)	Study the peripheral mobilization of MSC following corticosteroid administration in patients following liver transplantation or liver resection	NCT02557724	35	Published	N/A	Reduction in MSC migration following steroid administration
Wang, F	Phase 1 trial of MSC therapy to induce tolerance following liver transplantation	NCT01690247	50	Unknown	IV, details not described	Not published
Yang, Y	To determine if MSC are safe in acute rejection in ABO incompatible liver transplantation (Phase 1/2)	NCT02706132	15	Unknown	6 doses 1 × 10^6^/kg IV	Not published

## Concluding Remarks

Liver transplantation is a continually evolving field with transplant teams consistently pushing the boundaries to enable the scarce resource of a donor liver to confer greater benefit to increasingly larger numbers of patients. Cell therapy with MSC is an exciting treatment beginning to enter clinical trials that may allow the boundaries to be pushed even further for a greater patient benefit.

## Author Contributions

All authors listed have made a substantial, direct and intellectual contribution to the work, and approved it for publication.

## Conflict of Interest

The authors declare that the research was conducted in the absence of any commercial or financial relationships that could be construed as a potential conflict of interest.
